# Effects of Exogenous Abscisic Acid (ABA) on Carotenoids and Petal Color in *Osmanthus fragrans* ‘Yanhonggui’

**DOI:** 10.3390/plants9040454

**Published:** 2020-04-04

**Authors:** Yucheng Liu, Bin Dong, Chao Zhang, Liyuan Yang, Yiguang Wang, Hongbo Zhao

**Affiliations:** 1 Department of Ornamental Horticulture, School of Landscape Architecture, Zhejiang Agriculture and Forestry University, Lin’an 311300, China; liuyucheng27@163.com (Y.L.); dongbin@zafu.edu.cn (B.D.); zhangc@zafu.edu.cn (C.Z.); yangliyuan@126.com (L.Y.); wangyiguang1990@163.com (Y.W.); 2 Zhejiang Provincial Key Laboratory of Germplasm Innovation and Utilization for Garden Plants, Zhejiang Agriculture and Forestry University, Lin’an 311300, China; 3 Research Institute of Forestry, Chinese Academy of Forestry, Xiangshan Road, Beijing 100091, China

**Keywords:** *Osmanthus fragrans*, carotenoid, abscisic acid

## Abstract

*Osmanthus fragrans* is a well-known native plant in China, and carotenoids are the main group of pigments in the petals. Abscisic acid (ABA) is one of the products of the metabolic pathway of carotenoids. Application of ABA could affect pigmentation of flower petals by changing the carotenoid content. However, little is known about the effects of ABA treatment on carotenoid accumulation in *O. fragrans*. In this study, different concentrations of ABA (0, 150 and 200 mg/L) were spread on the petals of *O. fragrans* ‘Yanhonggui’. The petal color of ‘Yanhonggui’ receiving every ABA treatment was deeper than that of the control. The content of total carotenoids in the petals significantly increased with 200 mg/L ABA treatment. In the petals, α-carotene and β-carotene were the predominant carotenoids. The expression of several genes involved in the metabolism of carotenoids increased with 200 mg/L ABA treatment, including *PSY1*, *PDS1*, *Z-ISO1*, *ZDS1*, *CRTISO*, *NCED3* and *CCD4*. However, the transcription levels of the latter two carotenoid degradation-related genes were much lower than of the five former carotenoid biosynthesis-related genes; the finding would explain the significant increase in total carotenoids in ‘Yanhonggui’ petals receiving the 200 mg/L ABA treatment.

## 1. Introduction

*Osmanthus fragrans* is a favorite traditional flower in China because of its delightful flower color and scent. Carotenoids are the major pigments in the petals of *O. fragrans* [1]. Carotenoids produce many yellow, orange and red pigments in nature, including many fruits [2], vegetables, flowers [3], butterflies and crayfish [4]. In the petals, which mainly contain carotenoids, the kind and content of the carotenoids determine the color. In Marigold (*Calendula officinalis*), for example, the carotenoid content of the most pigmented varieties is approximately 100-fold that of white flowers [5]. In addition, carotenoid metabolism-related genes contribute to the color formation in these flowers, including biosynthesis- and degradation-related genes. In plants, carotenoids are derived from the 2-C-methyl-D-erythritol 4-phosphate (MEP) pathway in plastids [4]. Phytoene, formed by phytoene synthase (PSY), is the first committed step in the metabolic pathway of carotenoids. Then, the colorless phytoene is catalyzed by four enzymes, including phytoenedesaturase (PDS), ζ-carotene isomerase (Z-ISO), ζ-carotene desaturase (ZDS) and carotenoid isomerase (CRTISO), to produce lycopene. Lycopene is catalyzed by lycopene β-cyclase (LCYB) and lycopene ε-cyclase (LCYE) to form α-carotene; in contrast, β-carotene is catalyzed only by LCYB. Furthermore, α-carotene is converted to lutein by β-ring carotene hydroxylase (CHYB) and ε-ring carotene hydroxylase (CHYE), which is the end-product of the α-carotene branch. β-caroteneis catalyzed by CHYB twice to form zeaxanthin. Then, zeaxanthin is converted to neoxanthin by neoxanthin synthase (NSY). The isomers of violaxanthin and neoxanthinare cleaved by nine-cis-epoxy-carotenoid dioxygenases (NCEDs) to produce ABA [6,7,8]. 

Plant hormones play important roles in plant growth, development and secondary metabolism. Previous studies have shown that ethylene treatment can increase the content of carotenoids in different species, such as *Citrus sinensis* Osbeck [9], Satsuma mandarin (*Citrus unshiu Marc*.) [10] and papaya (*Carica papaya* cv. ‘Golden’) [11], because genes involved in biosynthesis of carotenoids, such as *PSY*, *LCYB* and *CHYB*, are upregulated by ethylene treatment [9]. In contrast, IAA treatment delays carotenoid accumulation in tomato [12]. For methyl jasmonate (MeJA), carotenoid accumulation was enhanced in Coriander (*Coriandrum sativum*) receiving MeJA treatment because of the increased transcripts level of *CsCHYE*, *CsPDS*, *CsZDS* and CsLCYE [13]. Other plant hormones, such as salicylic acid (SA), can also enhance the accumulation of carotenoids [14]. ABA regulates a wide range of plant growth and developmental processes [15,16] and mediates plant responses to environmental stresses [17,18]. In addition, the application of exogenous ABA has been shown to affect carotenoid content in *Lycopersicon esculentum*, as ABA treatment decreased the content of lutein but significantly increased the content of β-carotene. In two additional species of tomato, the application of ABA increased lutein, β-carotene and zeaxanthin in their leaves [19,20]. Correlated with the change in carotenoids, the expression of genes involved in the metabolic pathways of carotenoids was also influenced by ABA treatment. Recently, it has been shown that the expression of other carotenoid biosynthesis-related genes, such as *PSY*, could also be increased by ABA treatment [21]. In *O. fragrans*, the research are mainly about its flavor and color. Wang et al. found that *O. fragrans* could be divided into two clusters, an orange-red cluster and a yellowish-white cluster, respectively, in terms of the content of carotenoids and flavonoids in petals. The petals of the yellowish-white cultivars exhibited high contents of β-carotene, lutein and α-carotene, whereas the petals of the orange-red cultivars mainly contained β-carotene and α-carotene. The profound diversity in the total carotenoid concentrations of the two clusters was determined by the transcript levels of *OfCCD4* [22]. The main volatile organic compounds in petals of *O. fragrans* are linalool, α-ionone, β-ionone, and γ-decalactone, and 19 °C would promote the emission of a floral scent [23]. Studies have shown that the biosynthesis of volatile compounds in *O. fragrans* is caused by the OfCCD4 instead of OfCCD1 [24,25]. However, there is no report on ABA regulation of carotenoids in *O. fragrans*. Our purpose was to determine the effects of different ABA concentrations on carotenoids accumulation and the expression patterns of genes involved in carotenoids metabolism in the petals of *O. fragrans* ‘Yanhonggui’. This will help us have a better understanding of carotenoids accumulation in petals of *O. fragrans* under stress, because the production of ABA would increase when plants receive abiotic stress [26,27]. Furthermore, it would provide us with basic knowledge on breeding new cultivars of *O. fragrans* with deeper or lighter color petals. 

## 2. Results

### 2.1. Effect of ABA Treatment on the Petal Color

In order to understand the petals’ color change after ABA treatment, we used Color Reader to measure the phenotype of the petals’ a* (redness), b* (yellowness), L* (lightness), C* (chroma) and h* (hue-angle). The a* and b* values of ‘Yanhonggui’ petals receiving ABA treatment at S2 showed no difference compared to the control, and the L* values of petals receiving 150 mg/L ABA were higher than the other two treatments (0 and 200 mg/L ABA) (Table 1). At S3, the a* values were similar among treatments, while the L* value of the control was highest, followed by the 150 mg/L and 200 mg/L ABA treatments. There were no differences in h* values among every treatment. The L*, h*, C* and b* values were similar to the control, and the a* values for plants receiving the 150 mg/L ABA treatment were higher than the control but were similar to those receiving the 200 mg/L ABA treatment.

### 2.2. Effect of ABA Treatment on Carotenoid Composition of the Petals

**Figure 1:**
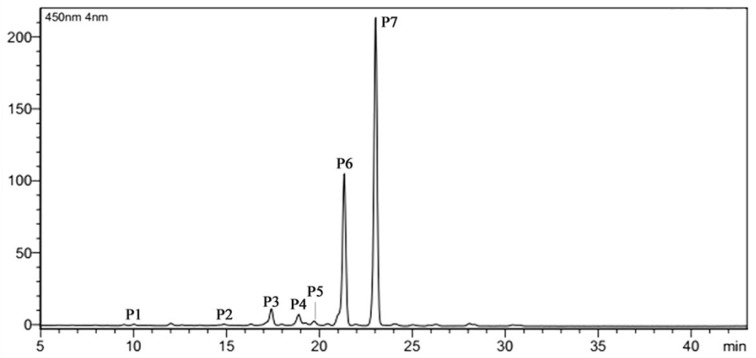
HPLC chromatograms of carotenoid pigments extracted from petals of ‘Yanhonggui’. P1: phytoene. P2: lutein. P3: internal standard β-apo-8′-carotenol. P4: unidentified carotenoid. P5: β-cryptoxanthin. P6: α-carotene. P7: β-carotene.

The carotenoids content in petals of ‘Yanhonggui’ were analyzed using the HPLC system. Carotenoids detected in ‘Yanhonggui’ petals included the internal standard β-apo-8′-carotenol (Figure 1, P3), phytoene (P1), lutein (P2), β-cryptoxanthin (P5), α-carotene (P6), β-carotene (P7), and an unidentified carotenoid (P4) based on their retention at S4 [22]. The total carotenoid content (TC) obtained with 200 mg/L ABA treatment was the highest, and the content of carotenoids in petals receiving the 150 mg/L ABA treatment was similar to that of the control, with 14,047.00 μg/g, 14,188.34 μg/g, and 15,328.64 μg/g dry weight (DW) content obtained for the tested treatments (control, 150 mg/L and 200 mg/L ABA treatment) (Table 2). The major carotenoids detected were α-and β-carotene, which accounted for more than 90% of the total carotenoids. Furthermore, with the 200 mg/L ABA treatment, β-carotene had the highest content (8887.31 μg/g DW); however, there was no significant difference between the 150 mg/L ABA treatment and the control of β-carotene. Similarly, the amount of α-carotene was highest for the 200 mg/L ABA treatment (5740.65 μg/g DW), followed by the 150 mg/L ABA treatment and the control (5089.04 μg/g and 4779.50 μg/g DW, respectively). Furthermore, the amounts of the other carotenoids were much lower than α- and β-carotene. Among these carotenoids, phytoene content showed no remarkable difference among all three treatments. For lutein, the content with ABA treatment was higher than that of the control. In contrast, the detected content of β-cryptoxanthin with the 150 mg/L ABA treatment was lower than that of the other two treatments. 

### 2.3. Effect of ABA Treatment on the Expression of Genes Involved in Carotenoid Metabolism in the Petals

**Figure 2:**
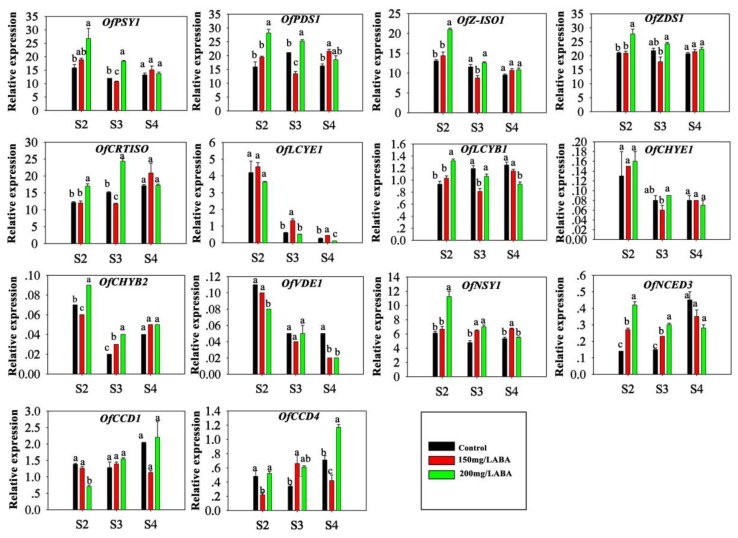
Expression levels of genes involved in carotenoid metabolism in ‘Yanhonggui’ petals receiving ABA treatments. Tukey’s multiple-range test was used, and least significant range analysis at 5% significance is shown by lowercase letters for the same stage for the control and treatments. All experiments were performed in triplicate. Data are shown as means ± SEM. Means followed by the same letter do not differ significantly.

Quantitative real-time PCR analysis of fourteen genes involved in carotenoid matabolism was performed. The *OfACT* gene was an internal control. At S2 and S3, high ABA treatment (200 mg/L ABA) increased the expression of the upstream genes *OfPSY1*, *OfPDS1*, *OfZDS1*, *OfCRTISO*, *OfCHYB2*, *OfNSY1* and *OfNCED3*, which are involved in the biosynthetic pathway of carotenoids, and the transcription levels of *OfZ-ISO* and *OfLCYB1* were only upregulated at S2 with the 200 mg/L ABA treatment. The expression of *OfCCD4* was upregulated at S3 and S4. Moreover, not all of the genes mentioned above were affected at other stages by ABA treatment. In contrast, expression of *OfCCD1* and *OfVDE* decreased with the 200 mg/L ABA treatment at S2. Unlike the gene expression patterns found with the 200 mg/L ABA treatment, the application of 150 mg/L ABA only increased the expression of several genes, such as *OfCHYB2* and *OfCCD4* at S3, *OfLCYE1* and *OfNSY1* at S3 and S4, and *OfNCED3* at S2 and S3. However, the transcript levels of other genes detected with the 150 mg/L ABA treatment were not affected and even decreased in some stages (Figure 2).

## 3. Discussion

Previous studies showed that the a* value was positively affected by TC [22]; in other words, the petals of cultivar which have deeper color contain more TC and the a* value was higher. In the present study, the a* value showed a significant difference at S4 but not S2 or S3 with ABA treatment. These results indicated that TC only changed at S4 significantly with ABA treatment in petals of ‘Yanhonggui’. So we only detected the carotenoid content at S4. The pattern of TC was consistent with the a* value pattern in petals receiving ABA treatment. The application of ABA increased the content of α-carotene, β-carotene and lutein in petals of ‘Yanhonggui’ (Table 2). Furthermore, α- and β-carotene were the main carotenoids, which confirms the petal color of ‘Yanhonggui’ [28]. In a previous study, it was reported that ABA treatment mainly increased the β-carotene and lutein content in the leaves of tomato [20]. In the fruit tissue of tomato, the β-carotene content was also increased by ABA treatment [19]. Lutein and β-carotene contents were also higher in ABA-treated plants than in control plants in Chinese cabbage and turnip [29,30]. Interestingly, different concentrations of ABA have different effects on the content of carotenoids. For pepper fruits, the main carotenoid, capsanthin, was increased by treatment with 150 mg/L ABA, while higher concentrations of ABA treatment reduced the content of capsanthin [31]. These results indicate that ABA treatments have positive effects on carotenoid accumulation. However, ABA treatment may have negative effects on carotenoids accumulation. The content of total carotenoids in tea plant (*Camellia sinensis*) flowers were reduced by ABA treatment [32]. Similar results were found in the callus of *Scutellaria baicalensis* after ABA treatment [33]. The effect of ABA treatment on carotenoids accumulation may vary among plant species. In pepper fruits, 150 mg/L ABA treatment promoted the accumulation of capsanthin, and it was 200 mg/L ABA treatment that significantly promoted carotenoid accumulation in the petals of ‘Yanhonggui’.

The genes involved in carotenoid metabolism—*OfPSY1*, *OfPDS1*, *OfZ-ISO1*, *OfZDS1* and *OfCRTISO, OfCCD4* and *OfNCED3*—were upregulated by ABA treatment in the present study. Consistent results were found in other species. In *S. baicalensis* [33] and *Brassica oleracea* [34], *CCD4* and *NCED* were also upregulated by ABA treatment; the expression of *OsPSY* and *OsNCED* in rice (*Oryza sativa*) [21] was higher with ABA treatment than in the control. Additionally, in *Brassica rapa*, *B. oleracea* [34], *Suaeda salsa* [35] and *Glycine max* [36], ABA upregulated transcript levels of *CCD1* and *CCD4*. PSY are the key enzymes indirectly regulating the biosynthesis of carotenoids that also contribute to phytoene—the first carotenoid produced in plants [6]. It has been indicated that abiotic stresses, including ABA, could regulate carotenoid accumulation via regulation of the expression of *PSY* [21,37]. Furthermore, ABA responsive elements (ABREs) have been found in the promoter of *OsPSY* [21] and *AhNCED1* in peanut (*Arachis hypogaea*) [38], *OfCCD1*, *OfCCD*4 [39] and *AtNCED* [40,41]—indicating that the expression of *CCD1*, *CCD4*, *NCED* and *PSY* could be induced by ABA because it has been reported that genes whose promoters contained ABREs could respond to ABA treatment in *Arabidopsis thaliana* [42,43]. The relative higher transcription of *OfPSY1*, *OfPDS1*, *OfZ-ISO1*, *OfZDS1* and *OfCRTISO* led to the higher content of precursor carotenoids. Then the higher transcripts level of *OfLCYB1*, which is the key gene involved in production of α- and β-carotene, caused a higher content of α- and β-carotene. While the precursor carotenoid detected was only phytoene and no lycopene was detected. These results were consistent with our previous study [22]. The little amount of precursor carotenoids could be caused by the downstream gene, *OfLCYB1*, in present study, because ABA treatment promotes the expression of it. Although the expression levels of *OfPSY1*, *OfPDS1*, *OfZ-ISO1*, *OfZDS1, OfCRTISO*, *OfCCD4* and *OfNCED* were upregulated, the transcription level of *OfCCD4* and *OfNCED* was much lower than that of *OfPSY*, *OfPDS1, OfZ-ISO1, OfZDS1* and *OfCRTISO*, which indicates that the content of produced carotenoids was much more than that of degraded carotenoids. Previous studies on carotenoids accumulation in different *O. fragrans* showed that, consistent with the higher carotenoids contents, *OfPSY1*, *OfPDS1*, *OfZ-ISO1*, *OfZDS1,* and *OfCRTISO* in the upstream pathways were highly expressed in the petals, and the expression profiles of *OfLCYB1* in petals were higher than those of *OfLCYE1*. Regarding the genes of the degradation pathway, the expression level of *OfCCD1* was negatively correlated with the concentration of carotenoids, whereas the expression levels of *OfNCED3* and *OfCCD4*, which were highly expressed in petals, showed no correlation with the TC content [22]. In the present study, the same genes mentioned above have similar expression patterns in petals. The expression of these genes was promoted with ABA treatment, especially the genes involved in carotenoids biosynthesis such as *OfPSY1* and *OfLCYB1*. Additionally, there is evidence of positive feedback regulation between *OsPSY3*, *OsNCEDs* and ABA treatment in rice. *OsPSY3* transcripts are up-regulated during increased abscisic acid (ABA) formation upon salt treatment and drought. The simultaneous induction of genes encoding 9-cis-epoxycarotenoid dioxygenases (NCEDs) is involved in the initial steps of ABA biosynthesis. Then, *OsPSY3* and the *OsNCEDs* are induced by ABA to promote ABA production [21]. Similarly, the application of ABA could indirectly regulate the metabolic pathway of carotenoids in *A. thaliana* [37]. According to the above conclusion and the results of the present study, we propose that there is a similar mechanism in the petals of ‘Yanhonggui’ treated with ABA. The application of ABA promoted the expression of genes whose promoters contained ABREs including *OfPSY* and *OfNCED*—the two key genes involved in ABA biosynthesis [21,44,45], and other genes like *OfPDS1*, *OfZ-ISO1*, *OfZDS1 OfCRTISO* and *OfLCYB1*. The higher expression of biosynthetic genes promotes the production of carotenoids, α-carotene and β-carotene, especially; on the other hand, the higher expression of *OfNCED3* could promote the biosynthesis of ABA. The newly biosynthesized ABA, cooperating with absorbed exogenous ABA, would promote the expression of *OfPSY1*, *OfPDS1*, *OfZ-ISO1*, *OfZDS1, OfCRTISO* and *OfNCED3*. Finally, the application of ABA significantly increased the total carotenoids in petals of ‘Yanhonggui’. ABA, genes including *OfPSY1*, *OfPDS1*, *OfZ-ISO1*, *OfZDS1, OfCRTISO* and *OfNCED3,* and carotenoids all formed a positive feedback loop in ‘Yanhonggui’ treated with ABA. As more carotenoids are contained in the petals, a deeper color is produced. It has been proven that pale flowers have fewer carotenoids than more deeply colored flowers [7]. Furthermore, the ABA-signaling pathway is a complicated network, which includes ABA receptors (ABARs) [46], type 2C phosphatases (PP2Cs) [47], ABRE-binding factors (ABFs) [48], and other proteins. Further investigation would be the measurement of ABA and the contribution of endogenous and absorbed exogenous ABA on the carotenoids metabolic pathway, and the mechanism of the ABA-signaling pathway on carotenoid metabolism to elucidate the mechanism of petal color change in *O. fragrans* treated with ABA. 

## 4. Materials and Methods

### 4.1. Plant Materials and Growth Condition 

**Figure 3:**
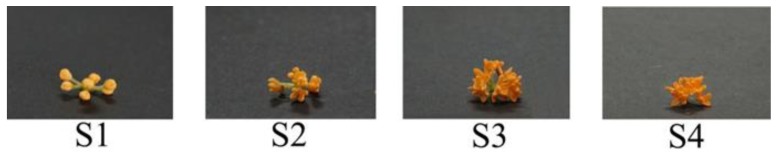
Flowering stages of ‘Yanhonggui’.

*O. fragrans* ‘Yanhonggui’ potted plants were grown under greenhouse conditions at Zhejiang A&F University and then were incubated at a constant temperature (25 °C) and relative humidity (70%) before the Linggeng stage (S1: flower buds are similar to round bells). The growth medium was a sterile mixture of peat: vermiculite: perlite (1:1:1). Samples were subjected to 12/12 hour light/dark periods until the treatment was completed. The light intensity inside the incubator was set to 5000 Lux by white fluorescent tubes. Then, 0 mg/L, 150 mg/L and 200 mg/L (±) ABA (Sigma-Aldrich, Shanghai, China) was spread onto the flowers every 24 h from the Linggeng stage (S1). Nine of *O. fragrans* ‘Yanhonggui’ potted plants with similar heights, and similar amounts of branches and buds were used in this present study. Each treatment contained three *O. fragrans* ‘Yanhonggui’ potted plants. Flower petals from different branches of same plant at the initial flowering stage (S2: flower petals start to expand), full flowering stage (S3: flower petals are fully open, and the anthers are yellow), and the late flowering stage (S4: flower petals are fully open, and the anthers are brown) [1] (Figure 3) were collected and mixed. At S2 and S3, mixed petals were randomly divided into two groups, part of the petals immediately frozen with liquid nitrogen, and then stored at −80 °C until they were used for gene expression analysis, and the rest was used for the measurement of flower color. At S4, mixed flower petals were randomly divided into three groups, and the first group of petals were immediately frozen with liquid nitrogen, and then stored at −80 °C until they were used for gene expression analysis; the second group of petals was directly frozen after collection by lyophilization in a freeze dryer and stored at −20 °C for carotenoids analysis. The third group of flower petals was used for the measurement of flower color. All nine plants were sampled at every stage.

### 4.2. Measurement of Flower Color

About 15 flowers at different branches from same plant were collected and mixed, and their petals were used for the measurement of flower color. Petals from every plant (all nine plants and three in each treatment), were measured respectively. The color of fresh petals was measured using lightness (L*), hue-angle (h*), and two chromatic components, a* and b*, of the CIEL*a*b*color coordinates using a Color Reader (CR-10; Konica Minolta Sensing Inc., Tokyo, Japan). L* values indicated lightness, and the h* values were calculated from tan^−1^(b*/a*) [49]. 

### 4.3. Carotenoids Extraction and Analysis

About 60 flowers from different branches of the same plants were mixed and their petals were used for carotenoid extraction and analysis. Petals from every plant (all nine plants and three in each treatment) were collected respectively. The extraction of carotenoids was performed following the method described by Delpinorius et al. (2014) [50], with some procedural improvements. After grinding with liquid nitrogen and drying by lyophilization in a dark room, 0.05 g dry sample was placed in a 10-mL vessel with 4 mL methanol. Next, 2.3 mL of a NaCl solution (10%, w/v) was added. The mixture was shaken for 15 min and cooled at 4 °C. The mixture was centrifuged at 2700× *g* for 3 min at 4 °C, and the organic layer was recovered. The aqueous phase was re-extracted with 2 mL of hexane: diethyl ether (3:1, v/v) for 10 min and centrifuged in the same conditions. The organic solutions were combined in a 5 mL glass tube and dried under a nitrogen stream at room temperature. The dry residue was saponified at room temperature for 24 h using 1.5 mL of a 6% KOH solution in methanol (w/v). After the addition of 1.5 mL of sodium chloride solution (10%, w/v), the mixture was placed in a freezer for 15 min. Then, 2.5 mL of hexane: diethyl ether (3:1, v/v) was added, and the mixture was vortexed and centrifuged at 433× *g* for 3 min (this step was repeated until the aqueous phase was colorless). The organic layers were combined, and the solvent was removed under a nitrogen stream. The residue was stored at −80 °C under nitrogen until HPLC analysis. 

### 4.4. Analysis of Carotenoids

The residue of carotenoids was dissolved in 1.5 mL methyl tert-butyl ether (MTBE) that contained β-apo-8′-carotenal as an internal standard (5 μg/mL) [51]. The carotenoid content analysis was performed by using a high-performance liquid chromatography (HPLC) system (Shimadzu Corporation, Kyoto, Japan). This system consists of an LC-20AT infusion pump, CTO-10AS VP column oven and SPD-M20A spectrophotometric detector. A C30-column (YMC Co. Ltd. Japan, 4.6 × 250 mm, 5 μm) was used at 25 °C, with a flow rate of 0.8 mL/min, and the data were recorded from 200 to 800 nm and measured at 450 nm. An amount of 10 μL analytes was injected. The elution solvents were solvent A (methanol) and solvent B (MTBE), and elution was performed as follows: 0 min, 0% B; 18min, 46% B; 35 min, 70% B; 37min, 70% B; 40min, 0% B; and 47min, 0% B.

### 4.5. RNA Extraction and cDNA Synthesis

About 60 flowers from different branches of the same tree were mixed and their petals were used for RNA extraction and analysis. Petals from every plant (all nine plants and three in each treatment), were collected and stored respectively. Samples from every plant were used for RNA extraction and cDNA synthesis. Total RNA was extracted using a NucleoSpin RNA Plant kit (TaKaRa, Dalian, China), and the cDNA was synthesized using a PrimeScript II Hi Fidelity RT-PCR kit (TaKaRa, Dalian, China).

### 4.6. Expression Analysis by Real-Time PCR

The primers used in RT-PCR reactions were developed by Zhang [28]. The expression level of *OfACT* was used as the reference [52]. The transcript levels of genes were analyzed by quantitative real-time PCR (RT-qPCR) using SYBRPremix Ex Taq II polymerase (TaKaRa, Dalian, China)and the Light Cycler 480 II Real Time System (Roche, Switzerland) according to the manufacturers’ instructions. Each 20 µL qRT-PCR reaction contained 10 µL SYBR R Premix Ex TaqTM II (Takara), 5 µL cDNA (100 ng/µL), 1.0 µL of 10 µM forward and reverse primer (designed using Primer Premier 5.0 software). The thermal cycling conditions were as follows: an initial denaturation of 30 s at 95 °C, followed by 40 cycles at 95 °C for 5 s and 60 °C for 30 s; then 95 °C for 5 s, 60 °C for 1 min and 95 °C for 15 s was used for dissociation curve analysis. The relative expression levels were calculated using 2-ΔCt [53]. Each statistical analysis was performed using IBM SPSS Statistics 19.0 (IBM, Armonk, NY, USA). The data were analyzed using a Tukey’s multiple-range test (*P* < 0.05) with three replicates.

## 5. Conclusion

In this research, *O. fragrans* ‘Yanhonggui’ were sprayed with different concentrations of ABA. The increased TC, α- and β-carotenes were due to the higher expression of *OfLCYB1* upon receiving 200 mg/L ABA treatment. The higher transcription levels of *OfPSY1*, *OfPDS1*, *Z-ISO1, OfCRTISO* were upregulated by ABA treatment in *O. fragrans* petals, providing the precursors to produce the main carotenes, α- and β-carotene. While the much lower expression levels of *OfNCED3* and *OfCCD4* caused resulted in the degraded carotenoids content being much lower than produced carotenoids content. As a result, the increased carotenoids content, especially α- and β-carotenes, was greater than that of the control, and led to the deeper color in *O. fragrans* petals.

## Figures and Tables

**Table 1:** Phenotype parameters of ‘Yanhonggui’ receiving different concentrations of ABA treatment.

Stage	Treatment	a*	b*	L*	C*	h*
S2	0 mg/L	0	35.60 ± 0.98a	52.35 ± 0.03a	63.00 ± 0.23b	63.32 ± 0.58a
	150 mg/L	150	33.50 ± 0.60a	54.77 ± 1.21a	67.33 ± 0.60a	64.20 ± 1.31a
	200 mg/L	200	35.57 ± 0.45a	51.93 ± 2.52a	63.13 ± 1.27b	62.96 ± 2.30a
S3	0 mg/L	0	38.70 ± 0.69a	47.95 ± 2.45a	61.75 ± 0.43a	61.64 ± 2.34a
	150 mg/L	150	38.80 ± 0.06a	50.55 ± 0.89a	60.00 ± 0.52a	63.73 ± 0.67a
	200 mg/L	200	41.20 ± 1.31a	49.20 ± 2.02a	58.67 ± 1.10b	64.17 ± 2.38a
S4	0 mg/L	0	42.35 ± 0.03b	48.80 ± 1.73a	59.15 ± 0.55ab	64.63 ± 1.29b
	150 mg/L	150	44.15 ± 0.55a	49.40 ± 1.33a	56.55 ± 0.61b	66.26 ± 1.36ab
	200 mg/L	200	44.20 ± 0.17a	55.43 ± 1.79a	60.83 ± 1.39a	70.91 ± 1.48a

S2: initial flowering stage. S3: full flowering stage. S4: late flowering stage. Tukey’s multiple-range test was used, and least significant range analysis at 5% significance is shown by lowercase letters for the same stage for the control and treatments. All experiments were performed in triplicate. Data are shown as means ± SEM. Means followed by the same letter do not differ significantly.

**Table 2:** Carotenoid content in the petals of ‘Yanhonggui’ receiving ABA treatments.

Content	0 mg/L ABA	150 mg/L ABA	200 mg/L ABA
P1	44.81 ± 0.91b	53.90 ± 2.53a	59.17 ± 1.15a
P2	33.53 ± 0.55c	53.92 ± 0.29a	43.96 ± 0.64b
P4	376.97 ± 0.36b	364.53 ± 0.38b	404.89 ± 7.80a
P5	185.05 ± 2.56a	150.90 ± 1.03b	192.67 ± 2.90a
P6	4779.50 ± 15.97c	5089.04 ± 64.07b	5740.65 ± 72.36a
P7	8627.15 ± 1.05b	8476.13 ± 98.27b	8887.31 ± 27.09a
TC	14,047 ± 13.07b	14,188.43 ± 165.24b	15,328.64 ± 95.05a

Tukey’s multiple-range test was used, and least significant range analysis at 5% significance is shown by lowercase letters for the same stage for the control and treatments. All experiments were performed in triplicate. Data are shown as means ± SEM. Means followed by the same letter do not differ significantly.
